# T cell immunity to seasonal Influenza A and H5N1 viruses in laboratory workers receiving annual seasonal Influenza vaccines

**DOI:** 10.3389/fimmu.2025.1718805

**Published:** 2025-12-18

**Authors:** Joel Sop, Tyler P. Beckey, Lizeth Gutierrez, Li Zhang, Kelly A. Gebo, Kellie N. Smith, Joel N. Blankson

**Affiliations:** 1 Department of Medicine, Johns Hopkins Medicine, Baltimore, MD, United States; 2 Bloomberg-Kimmel Institute for Cancer Immunotherapy, Johns Hopkins Medicine, Baltimore, MD, United States; 3 Sidney Kimmel Comprehensive Cancer Center, Johns Hopkins University, Baltimore, MD, United States; 4 Department of Molecular and Comparative Pathobiology, Johns Hopkins Medicine, Baltimore, MD, United States

**Keywords:** H5N1 (avian influenza), T cell epitope, SARS - CoV - 2, T cell, vaccine

## Abstract

**Background:**

Emerging threats such as highly pathogenic influenza strains like H5N1 emphasize the need for vaccines that induce cross-reactive immunity against conserved epitopes. Existing influenza vaccines primarily elicit strain-specific responses, leaving gaps in protection against pandemic subtypes. This study aimed to evaluate T cell responses to seasonal influenza A and H5N1 and compare them to SARS-CoV-2 specific T cell responses to understand differences shaped by distinct exposure histories and vaccination strategies.

**Methods:**

T cell responses were assessed in 41 laboratory workers who received annual seasonal influenza vaccines using ELISpot to quantify responses to peptide pools derived from influenza (H1N1 hemagglutinin [HA], H3N2 HA, H5N1 HA, matrix protein 1 [MP1], nucleoprotein [NP]) and SARS-CoV-2 (spike [S2S], nucleocapsid [S2N]). Ten-day expansion assays were used to evaluate functional cross-reactivity between H1, H3, and H5 HA. Intracellular cytokine staining was performed to assess antigen-specific T cell functionality. We used the IFN-γ ELISpot assay and intracellular cytokine staining to evaluate T cell responses to H5N1 HA peptides and assessed cross-reactivity and functional similarity in H1N1 HA-expanded cells.

**Results:**

The percentage of individuals with effector T cell responses to influenza peptide pools, was markedly lower than the percentage of individuals with S2S-specific T cells. However, HA-specific memory cells that cross-recognized H1, H3, and H5 HA were present in many individuals. T cells expanded with H1 or H5 HA proteins cross-recognized homologous epitopes in the 2 proteins and cytokine production profiles were comparable between H1- and H5-expanded T cells.

**Conclusion:**

These results highlight the potential for influenza vaccines to elicit cross-reactive immunity against H5N1 viruses. These findings also demonstrate differences between T cell responses to influenza and SARS-CoV-2, highlighting distinct immune profiles that could inform future vaccine strategies.

## Introduction

Influenza viruses remain a major global health challenge, causing significant morbidity and mortality during seasonal epidemics and posing substantial risks through periodic pandemics (1). Influenza A viruses (IAVs), including highly pathogenic avian strains like H5N1, have demonstrated the potential for cross-species transmission and zoonotic infections (2). H5N1 is particularly concerning due to its high global case fatality rate, often exceeding 50% (3, 4), though lower mortality rates have been reported in recent studies in the United States (5–8). The World Health Organization (WHO) and other global health authorities have identified H5N1 as a pandemic threat, emphasizing the need for improved countermeasures (9).

Influenza vaccines currently rely on inducing strain-specific immunity against surface glycoproteins specifically, hemagglutinin (HA) and neuraminidase (NA) (10). However, these antigens are highly variable due to antigenic drift and shift, necessitating frequent vaccine updates and offering limited protection against novel subtypes like H5N1 (11). In contrast, internal viral proteins such as nucleoprotein (NP) and matrix protein 1 (MP1) are more conserved and recognized by T cells, making them promising targets for eliciting cross-reactive immune responses (12–15). T cells play a critical role in mediating immunity to viral infections by recognizing conserved epitopes, thereby contributing to cross-reactive responses that can bridge immunity across subtypes (16–25). Previous studies have demonstrated that cross-reactive T cells, particularly those targeting conserved epitopes, can provide protection against antigenically diverse influenza strains, including those with pandemic potential (26–29). Pre-existing T cells targeting these conserved regions have been correlated with protection from severe disease during pandemics such as the 2009 H1N1 outbreak (30–33).

The COVID-19 pandemic caused by SARS-CoV-2 has also underscored the importance of understanding immune responses to rapidly evolving pathogens (34–37). SARS-CoV-2 vaccines have demonstrated robust T cell responses (37–41), providing an opportunity to compare the functionality of T cell responses elicited by influenza and SARS-CoV-2 vaccines. In both cases, T cells have shown the capacity to recognize conserved epitopes and protect against severe disease (22, 42–48). However, the extent to which these responses differ, particularly in terms of polyfunctionality and cross-reactivity, remains poorly understood.

In this study, we compared the frequency of influenza-specific and SARS-CoV-2-specific T cells in a cohort of laboratory workers who receive annual seasonal influenza vaccinations and at least one COVID mRNA vaccine. Using ELISpot, intracellular cytokine staining, and functional expansion assays, we evaluated T cell functionality, cross-reactivity, and the targeting of conserved epitopes. The findings have implications for the design of vaccines that provide broad protection against both seasonal and pandemic viruses.

## Methods

### Study participants

This study was approved by the Institutional Review Board (IRB) of Johns Hopkins University (IRB00027183). Written informed consent was obtained from all participants prior to their enrollment. The study cohort included 41 laboratory workers (17 men and 24 women, between 20 and 60 years of age), who receive mandatory annual seasonal influenza vaccinations. The participants had received a median of 4 influenza vaccines at Johns Hopkins, and most reported receiving annual vaccines for many years prior to working at the university. None of the participants worked with H5N1 viruses or peptides. Blood was drawn before the seasonal influenza vaccine in 2024 to determine baseline T cell responses. Thus, the participants were a year removed from their last influenza vaccine. In a subset of participants, blood was also drawn after the influenza vaccine to compare pre and post vaccine responses. The median time between vaccination and the second blood draw was 22 days (range 13–51 days, interquartile range of 22 days). Detailed characteristics of the participants are provided in **Supplementary Table 1**.

### ELISpot assay

An interferon-gamma (IFN-γ) ELISpot assay was performed using peptide pools derived from influenza and SARS-CoV-2 antigens. The peptide pools included H1N1 HA (Influenza A virus/New York/18/2009 pandemic strain), H3N2 HA (Influenza A virus/New York/384/2005), H5N1 HA Thailand (A/Thailand/1(KAN-1)/2004), nucleoprotein (NP from H1N1, Influenza A virus/New York/348/2003), matrix protein 1 (MP1 from H1N1, Influenza A virus/California/04/2009 pandemic strain), SARS-CoV-2 spike (S2S), and SARS-CoV-2 nucleocapsid (S2N), all obtained from BEI Resources. Each peptide pool was used at a final concentration of 10 μg/mL and incubated with 200,000 cells/well in RPMI media supplemented with 10% fetal bovine serum for 20 hours at 37°C, following the manufacturer’s instructions as previously described (46, 49). Cells were added to each well, and the plates were incubated for 20 hours with the different sets of Influenza and SARS-CoV2 peptide pools. For each condition, two replicates were performed.

An additional IFN-γ ELISpot assay was conducted using 94 individual peptides spanning the H5N1 HA protein, also obtained from BEI Resources. This peptide pool was used at a final concentration of 1 μg/mL, with PBMCs plated at 200,000 cells/well and cultured with individual peptides for 20 hours under identical conditions. Two replicates were performed for each peptide.

A second additional IFN-γ ELISpot assay was conducted using peptide pools spanning the H3N2 HA (Influenza A virus/New York/384/2005), H5N1 HA Thailand (A/Thailand/1(KAN-1)/2004), and H5N1 HA Vietnam (A/Vietnam/1203/2004) proteins. Each peptide pool was used at a final concentration of 1 μg/mL and incubated with 200,000 cells/well under the same culture conditions as described above. Two technical replicates were performed for each condition.

ELISpot plates were analyzed by an independent investigator using the AID iSpot Spectrum and vendor-supplied software. The software quantified IFN-γ spot-forming units (SFUs) per well. SFU/million cells was calculated by adjusting the number of spots per well with the appropriate dilution factor. For each donor, the stimulation index (SI; fold-change over DMSO-treated controls) was calculated by dividing the SFU of each peptide condition by the SFU of the corresponding DMSO control. A positive response was defined as a mean SFU of ≥30 and SI> 3. All raw data for the ELISpot assays, are provided in **Supplementary Table 2**.

Protein sequence alignment between H5N1 HA Thailand (A/Thailand/1(KAN-1)/2004), H1N1 HA (Influenza A virus/New York/18/2009 pandemic strain) and H3N2 HA (Influenza A virus/New York/384/2005) was performed using VectorBuilder with EBLOSUM62 matrix (**Table 1**; **Supplementary figure 3**).

**Table 1:** Sequence homology analysis of H5N1 HA peptides recognized by study participants.

Participant ID	Targeted H5N1 HA peptide	H5N1 HA, Thailand	H1N1 HA, New York	% identity to H5 HA peptide	H3N2 HA, New York	% identity to H5 HA peptide
HD54	57	333 LRNSPQRERRRKKRGLF 349	335 LRN**V**P**SIQ—S**RGLF 347	47.1	336 **M**RN**V**P**–**E**–KQT**RG**I**F 348	41.2
	58	339 RERRRKKRGLFGAIAGF 355	341 **IQ—S**RGLFGAIAGF 353	58.8	341 **-**E**–KQT**RG**I**FGAIAGF 354	58.8
	59	345 KRGLFGAIAGFIEGGWQ 361	343 **S**RGLFGAIAGFIEGGW**T** 359	88.2	344 **T**RG**I**FGAIAGFIE**N**GW**E** 360	76.5
	89	524 LESIGIYQILSIYSTVA 540	522 LES**TR**IYQIL**A**IYSTVA 538	82.4	523 L**K**S**-**G**-**Y**KDW**IL**W**I**-**S**-F**A 537	52.9
	94	553 SLWMCSNGSLQCRICI 568	551 S**F**WMCSNGSLQCRICI 566	93.8	551 **IM**W**A**C**QK**G**NIR**C**N**ICI 566	43.8
HD140	36	207 YQNPTTYISVGTSTLNQ 223	209 YQN**ADA**Y**VF**VGTS**RYSK** 225	47.1	211 Y**A**Q**-ASGR**I**T**V**S**T**KR-S**Q 226	35.3
	59	345 KRGLFGAIAGFIEGGWQ 361	343 **S**RGLFGAIAGFIEGGW**T** 359	88.2	344 **T**RG**I**FGAIAGFIE**N**GW**E** 360	76.5
	94	553 SLWMCSNGSLQCRICI 568	551 S**F**WMCSNGSLQCRICI 566	93.8	551 **IM**W**A**C**QK**G**NIR**C**N**ICI 566	43.8

Amino acid sequences and percent identity comparison between H5N1 HA peptides that elicited positive T cell responses (SFU ≥30) in participants HD54 and HD140 and the corresponding regions in H1N1 HA (A/New York/18/2009) and H3N2 HA (A/New York/384/2005) proteins. Bold letters indicate amino acid differences between H5N1 and comparison sequences. Dashes represent gaps in sequence alignment.

### T cell expansion culture assays and polyfunctionality analysis

PBMCs were cultured in R10 media with 10 IU/mL IL-2 and 10 μg/mL peptide pools (H1N1 HA, H3N2 HA, H5N1 HA, MP1, S2S) for 10 days. Cell cultures ranged from 12 to 64 million PBMCs per condition. Media replenishment occurred on days 3 and 7 by replacing half the culture medium with RPMI media with 10% fetal bovine serum (R10 media) containing 10 IU/mL IL-2. After 10 days, the cells were washed and replated in R10 media with 10 U/mL IL-2 and rested for at least 8 hours in a 37°C incubator before stimulation. Restimulation was performed with the same peptide pools (10 μg/mL) in the presence of protein transport inhibitors (GolgiPlug, BD Biosciences, 1 μg/mL; GolgiStop, BD Biosciences, 0.7 μg/mL) and co-stimulatory antibodies against CD28 (BD Biosciences) and CD49d (BD Biosciences). After a 16-hour incubation at 37°C, the cells were washed and stained with the Zombie NIR Fixable Viability Kit (BioLegend) and then surface-stained with antibodies against CD3 (Pacific Blue, BioLegend), CD4 (PerCP-Cy5.5, BioLegend), and CD8 (BV-605, BioLegend). Following surface staining, cells were fixed and permeabilized and subsequently stained intracellularly with antibodies against TNF-α (PE-Cy7, BD Biosciences), IFN-γ (APC, BD Biosciences), and IL-2 (PE, BioLegend). Flow cytometry was performed using a BD FACS LSR Fortessa flow cytometer, and at least 100,000 events were collected within the lymphocyte gate. Data were analyzed using FlowJo software (version 10.10.0) to identify cytokine-producing, antigen-specific T cells. The gating strategy used is shown in **Supplementary Figure 1**.

The polyfunctional profiles of antigen-specific T cells were analyzed using Pestle (version 2.0) and SPICE (version 6.1). Data from intracellular cytokine staining (ICS) and Boolean gating were processed in FlowJo and subsequently exported for further visualization and analysis in SPICE. GraphPad Prism software (version 10.4.1) was used to generate statistical analyses of the polyfunctional T cell data.

### Identification of antigen-specific TCRs using the FEST assay

The functional expansion of specific T cells (FEST) assay was employed to identify antigen-specific T cell receptors (TCRs). This assay quantitatively sequences the CDR3 region of the beta chain of TCRs from T cells cultured with peptide antigens, allowing the identification of expanded, antigen-specific clones (50). The antigen specificity of TCRs identified via FEST has been previously validated by cloning the receptors into Jurkat cell lines (51). This supports the high accuracy of this assay in identifying bona fide antigen-specific TCRs. In a prior study (48), the FEST assay was applied to CD8+ T cell-depleted PBMCs from 10 participants to assess TCR responses to H1N1 HA and ancestral SARS-CoV-2 spike peptide pools. In addition, the FEST assay was applied to CD4+ T cells from 3 healthy donors cultured with H1N1 and H5N1 HA peptide pools in triplicate, enabling the identification of mono-reactive and cross-reactive antigen-specific TCR clonotypes to these influenza strains. PBMCs were cultured in triplicate at a density of 2 million cells per well in a medium containing IMDM, 5% human AB serum, 10 IU/mL IL-2, 50 μg/mL gentamicin, and 1 μg/mL peptide pool (H1 HA, H5 HA and S2S). Media replenishment occurred on days 3 and 7, with half of the media replaced with fresh culture medium. After 10 days of culture, cells were harvested, and DNA was extracted using the QIAmp Micro DNA Kit (QIAGEN) according to the manufacturer’s instructions. TCR sequencing (TCR-Seq) was performed at the Johns Hopkins FEST and TCR Immunogenomics Core Facility (FTIC) using the AmpliSeq for Illumina TCR Beta-SR panel. Sequencing was conducted on an Illumina MiSeq platform with dual indexes after pooling samples. Preprocessing steps included removing nonproductive TCR sequences, aligning, and trimming nucleotide sequences to retain only the CDR3 region. Sequences were excluded if they did not start with cysteine (C) or end with phenylalanine (F) or tryptophan (W), or if they contained fewer than seven amino acids in the CDR3 region. The median number of TCRs analyzed per participant was 15,522.5, ranging from 1,930 to 34,142 receptors.

Processed data files were then uploaded to our publicly available MANAFEST analysis web application (http://www.stat-apps.onc.jhmi.edu/FEST/) to bioinformatically identify antigen-specific T cell clonotypes. A positive TCR response to H1N1 HA or ancestral spike peptides was defined based on specific criteria, including a mean frequency threshold of greater than 0.1% for each of the three replicates, with at least two replicates having a frequency greater than 0.1%, and the mean frequency being at least 5-fold greater than the mean frequency of wells containing DMSO alone. A response was identified if all three conditions were met. All individual TCRs analyzed in all the FEST assays are detailed in **Supplementary Table 3**.

### Statistical analysis

Statistical analyses were performed using GraphPad Prism version 10.4.1 (GraphPad Software, San Diego, CA). For all tests, a two-sided p-value < 0.05 was considered statistically significant. Specific tests used were as follows.

ELISpot comparisons: Wilcoxon matched-pairs signe-rank test was used to compare paired pre- and post- vaccination responses for H1 HA, H3 HA, and H5 HA.

FEST TCR frequency comparison: Wilcoxon matched-pairs signed-rank test was used to compare the percentage of H1 HA-specific versus S2S-specific TCR clonotyoes within the same participants (n=10).

Cross-reactivity expansion: Friedman test with Dnn’s multiple comparisons correction was used to compare responses across multiple peptide pools (H1 HA, H3 HA, H5 HA) within the same donors after expansion.

Polyfunctionality analysis: Spice version 6,1 was used to generate pie charts. Comparisons of cytokine profiles between H1 HA and S2S conditions were performed using permutation test as implemented in SPICE WITH 10,000 iterations.

No corrected for multiple comparisons was applied to exploratory analyses unless otherwise stated. Effect sizes are reported as median differences with 95% confidence intervals where applicable.

## Results

### T cell responses to influenza proteins are markedly lower than responses to SARS-CoV-2 proteins, and influenza vaccination significantly boosts HA-specific T cell responses

**Figure 1:**
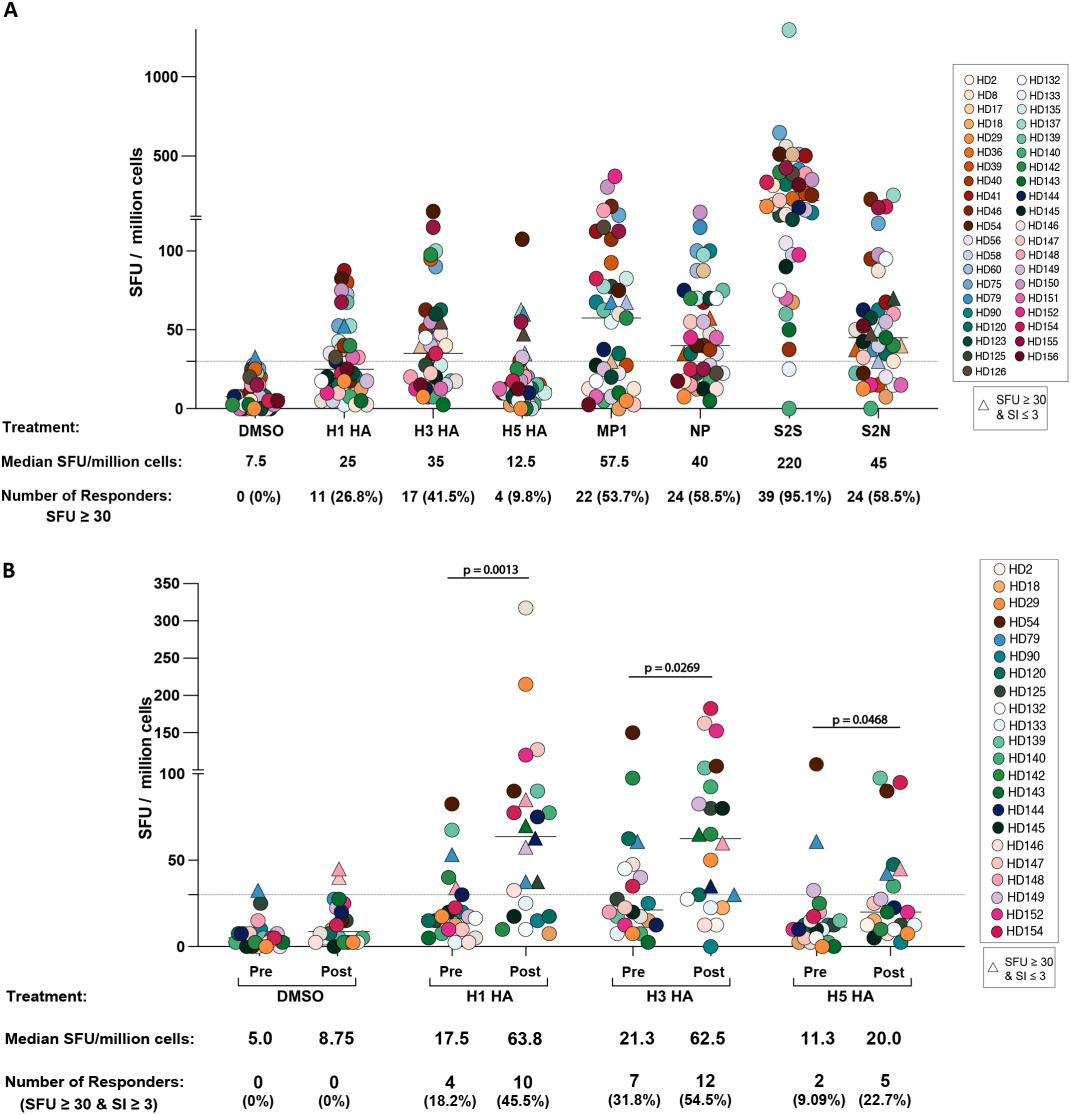
T cell responses to influenza and SARS-CoV-2 peptide pools in laboratory workers. **(A)** The number of SFU per million PBMCs in response to different peptide pools (H1N1 HA, H3N2 HA, H5N1 HA, MP1, NP, S2S, S2N) or the DMSO dilutant alone is shown for 41 healthcare and laboratory workers who receive annual seasonal influenza vaccinations. The dotted horizontal line represents the threshold for positive responses (SFU ≥30). **(B)** The number of SFU per million PBMCs in response to different peptide pools (H1N1 HA, H3N2 HA, H5N1 HA) or DMSO is shown for 22 participants before and after seasonal influenza vaccination. The dotted horizontal line represents the threshold for positive responses (SFU ≥30). ∗p < 0.05, ∗∗p < 0.01.

T cell responses were analyzed with the ELISpot assay in 41 healthy donors prior to the receipt of the annual seasonal influenza vaccine. A higher percentage of participants responded to the conserved internal influenza proteins MP1 (53.7%) and NP (58.5%) compared to the HA proteins of H1N1 (26.8%), H3N2 (41.5%), and H5N1 (9.8%). Responses to SARS-CoV-2 antigens were more robust, with 95.1% of participants responding to the spike protein (S2S) and 58.5% responding to the nucleocapsid protein (S2N) (**Figure 1A**).

The impact of seasonal influenza vaccination on HA-specific responses was assessed in 22 participants. Prior to vaccination, the percentage of participants responding to H1, H3, and H5 HA was 18.2%, 31.8%, and 9.09%, respectively. After vaccination, these response rates significantly increased to 45.5% (p = 0.0013), 54.5% (p = 0.0269), and 22.7% (p = 0.0468), respectively, with corresponding increases in the frequency of responding T cells. Notably, while the median T cell responses to H1 HA and H3 HA exceeded the SFU ≥ 30 and SI > 3 threshold post-vaccination, the median response to H5 HA, despite an increase, remained below this threshold (**Figure 1B**).

In order to compare the frequency of T cell responses to different H5 HA proteins, we performed ELISpot assays with peptides from H5N1 HA Thailand (A/Thailand/1(KAN-1)/2004), and H5N1 HA Vietnam (A/Vietnam/1203/2004) proteins with 28 participants. A greater proportion of participants responded to H3N2 HA (57.1%) compared to H5N1 HA Thailand (10.7%) and H5N1 HA Vietnam (7.1%, **Supplementary figure 2**).

### H1 HA-specific T cells exhibit strong expansion and polyfunctional cytokine profiles

To investigate the functional capacity of influenza-specific memory T cells and compare them to SARS-CoV-2-specific responses, PBMCs from 10 healthy donors were cultured with H1 HA or S2S peptide pools for 10 days, followed by 16-hour restimulation with the same peptide pools. Cytokine production was assessed using intracellular cytokine staining and flow cytometry.

**Figure 2:**
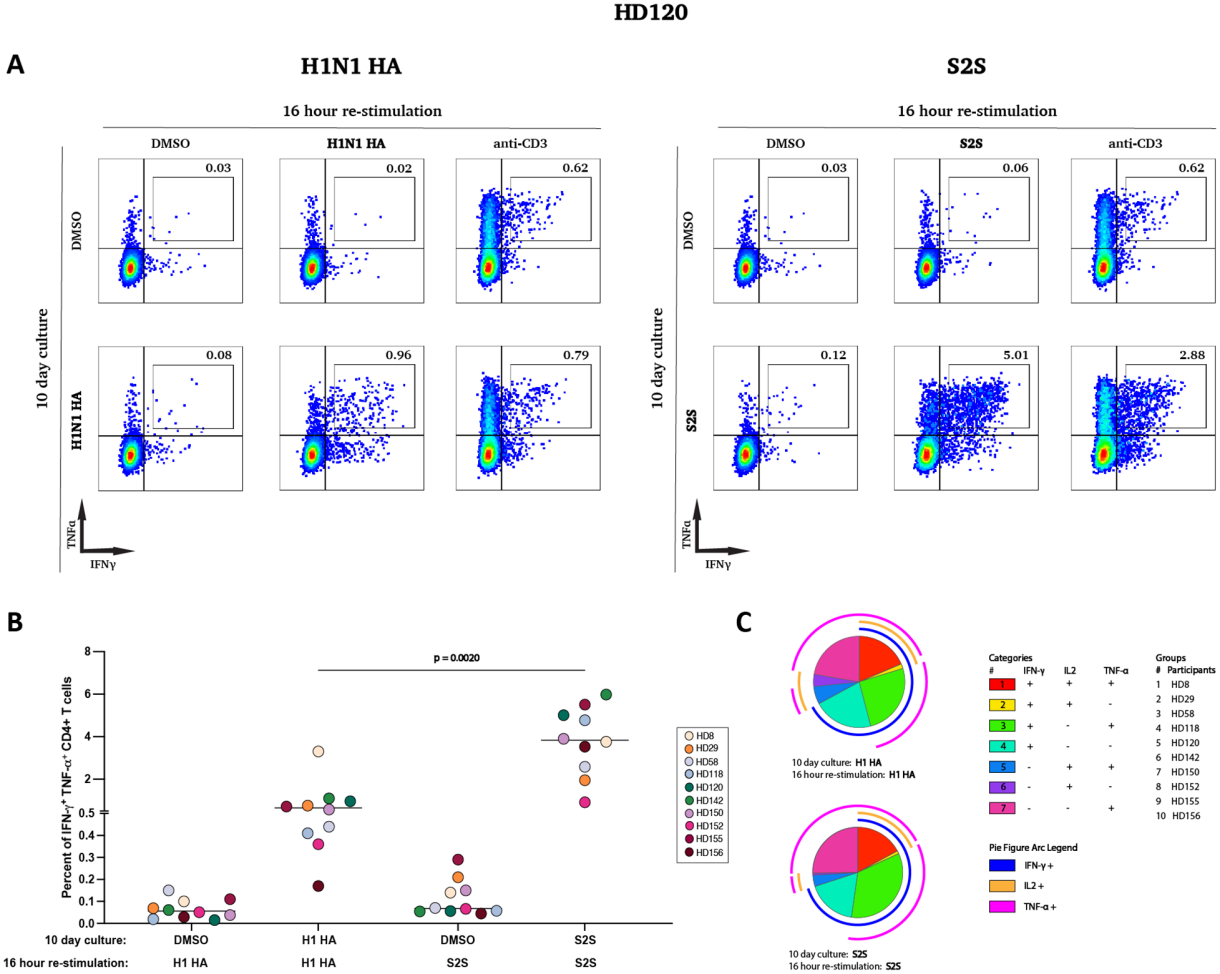
Functional characterization of antigen-specific CD4+ T cell responses following *in vitro* expansion. **(A)** PBMCs from one participant (HD120) were pre-cultured for 10 days with either DMSO (top row) or H1N1 HA (left bottom row) & S2S peptides (right bottom row). After expansion, cells were stimulated for 16 hours with DMSO, H1N1 HA peptides, S2S peptides, or anti-CD3 antibodies and stained for IFN-γ and TNF-α expression. The percentages in each quadrant indicate the proportion of cells expressing the respective cytokines. The same set of cells were pre-cultured for 10 days with DMSO before re-stimulation with either DMSO, H1N1 HA, S2S or anti-CD3. Thus, identical plots are shown for DMSO and anti-CD3 restimulation for cells pre-cultured with DMSO as comparators for cells pre-cultured with H1N1 HA or S2S. **(B)** The percentage of IFN-γ+ TNF-α+ CD4+ T cells was measured in 10 participants after a 10-day pre-culture and 16-hour restimulation with H1N1 or S2S peptide pools, with DMSO as the negative control. ∗∗p < 0.01. **(C)** Polyfunctionality of antigen-specific T cells was assessed using Pestle v2.0 and SPICE 6 v6.1. An antigen-specific T cell can produce one or multiple cytokines simultaneously. In this study, we evaluated the production of IFN-γ, IL-2, and TNF-α, analyzing seven possible cytokine expression profiles while excluding cells that did not produce any of these cytokines (IFN-γ- IL-2- TNF-α-).

Restimulation of H1 HA-expanded T cell lines resulted in robust cytokine production, as evidenced by a significant increase in CD4+ T cells co-expressing IFN-γ and TNF-α compared to cells that were cultured for 10 days in the presence of DMSO (median 0.64% *vs*. 0.056%). S2S-expanded T cells exhibited stronger responses, with median frequencies of IFN-γ+ TNF-α+ cells reaching 3.83% compared to 0.068% for cells that were cultured for 10 days in the presence of DMSO (p = 0.0020, **Figures 2A, B**).

A polyfunctionality analysis revealed distinct cytokine profiles for H1N1 HA- and S2S-specific T cells. Both groups exhibited comparable frequencies of highly functional IFN-γ+ TNF-α+ IL-2+ subsets (13.9% for H1N1 HA *vs*. 16.7% for S2S). However, S2S-specific T cells contained a significantly higher proportion of IFN-γ+ TNF-α+ IL-2- subsets (31.2% *vs*. 24.0%, p = 0.0039), while H1N1 HA-specific T cells displayed a higher proportion of IFN-γ- TNF-α- IL-2+ subsets (3.07% *vs*. 0.26%, p = 0.0156). There were no significant differences between other subsets (**Figure 2C**).

### Lower frequency of H1 HA-specific T cell receptors compared to S2S-specific TCRs

The frequency of CD4+ T cell receptors (TCRs) specific for H1 HA and SARS-CoV-2 spike (S2S) was compared using the FEST assay, which identifies antigen-specific TCRs by sequencing the CDR3 region of the beta chain in expanded T cells (52). CD8+ T cell-depleted PBMCs from 10 healthy donors were stimulated with H1N1 HA or S2S peptide pools for 10 days before TCR sequencing in a prior study (46).

**Figure 3:**
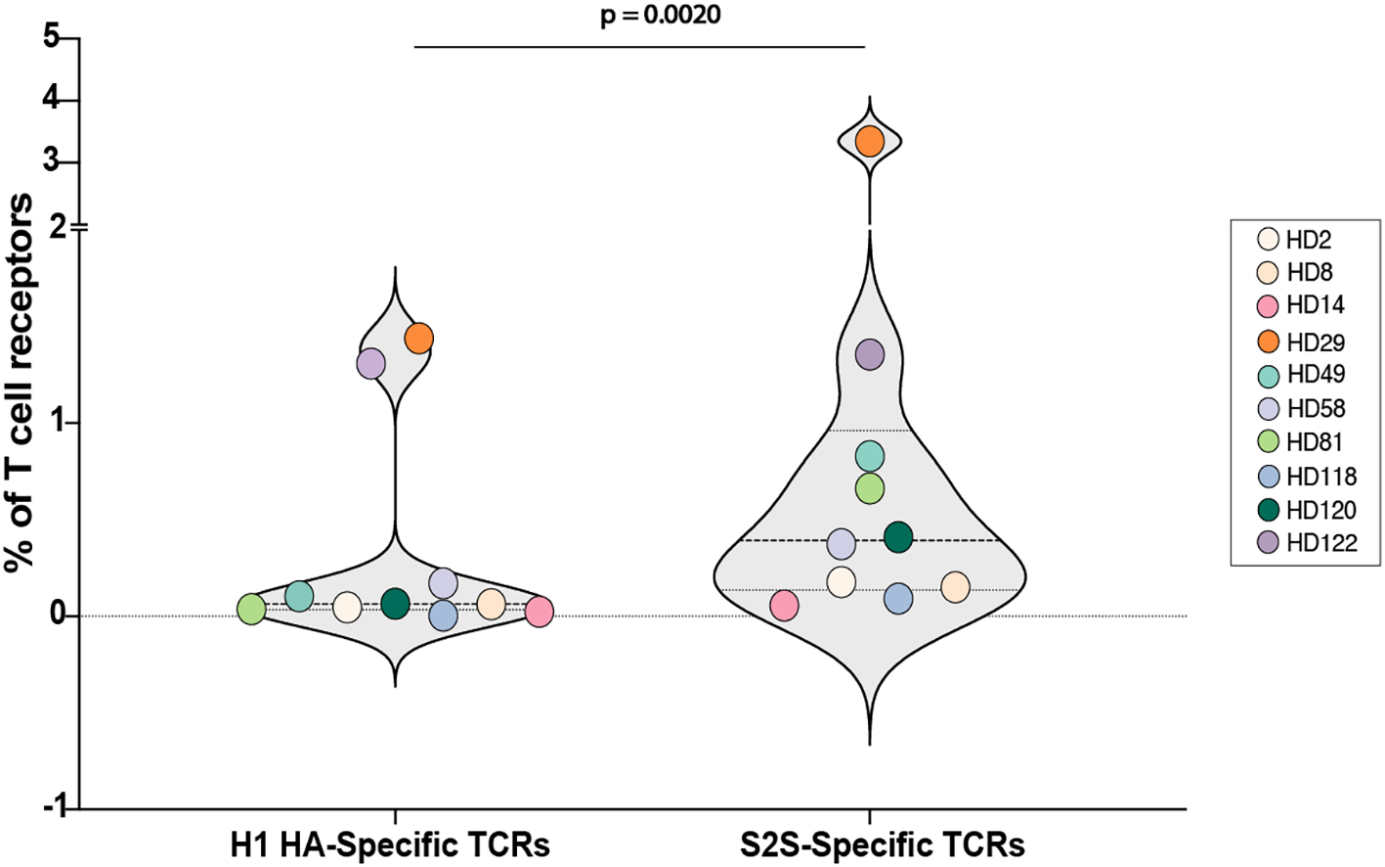
Identification of H1N1 HA- and S2S-specific memory TCRs using the FEST assay. After culture, TCR Vβ CDR3 sequencing was performed to identify antigen-specific memory T cells that expanded in response to H1 HA and S2S peptide pools (FEST assay). Antigen-specificity was defined by the functional expansion of the same CD4+ TCR clonotypes in response to pooled H1 HA and S2S peptides. Three technical replicates were run for each peptide culture for each of the 10 participants. The percentage of antigen-specific TCRs that were H1 HA-specific versus S2S-specific for all 10 participants is shown. ∗∗p < 0.01.

The analysis revealed a difference in the frequency of antigen-specific TCRs. After 10 days of culture in the presence of the respective antigens, S2S-specific TCRs constituted a median of 0.39% of the total sequenced TCRs. This was significantly higher than the 0.064% median frequency observed for H1 HA-specific TCRs (p =0.002, **Figure 3**). Across the cohort, all donors demonstrated this trend, highlighting the greater abundance of S2S-specific TCRs compared to those specific for H1 HA after antigen-specific expansion.

### H1 and H5 HA-specific memory T cells exhibit robust cross-reactive responses with comparable polyfunctional profiles

To investigate the cross-reactivity of H1 and H5 HA-specific memory T cells with other influenza HA subtypes, PBMCs from 14 healthy donors were stimulated with H1 or H5 HA peptide pools for 10 days to generate antigen-specific T cell lines. These lines were then restimulated for 16 hours with peptide pools from H1, H3, or H5 HA, and cytokine production was measured using intracellular cytokine staining and flow cytometry.

**Figure 4:**
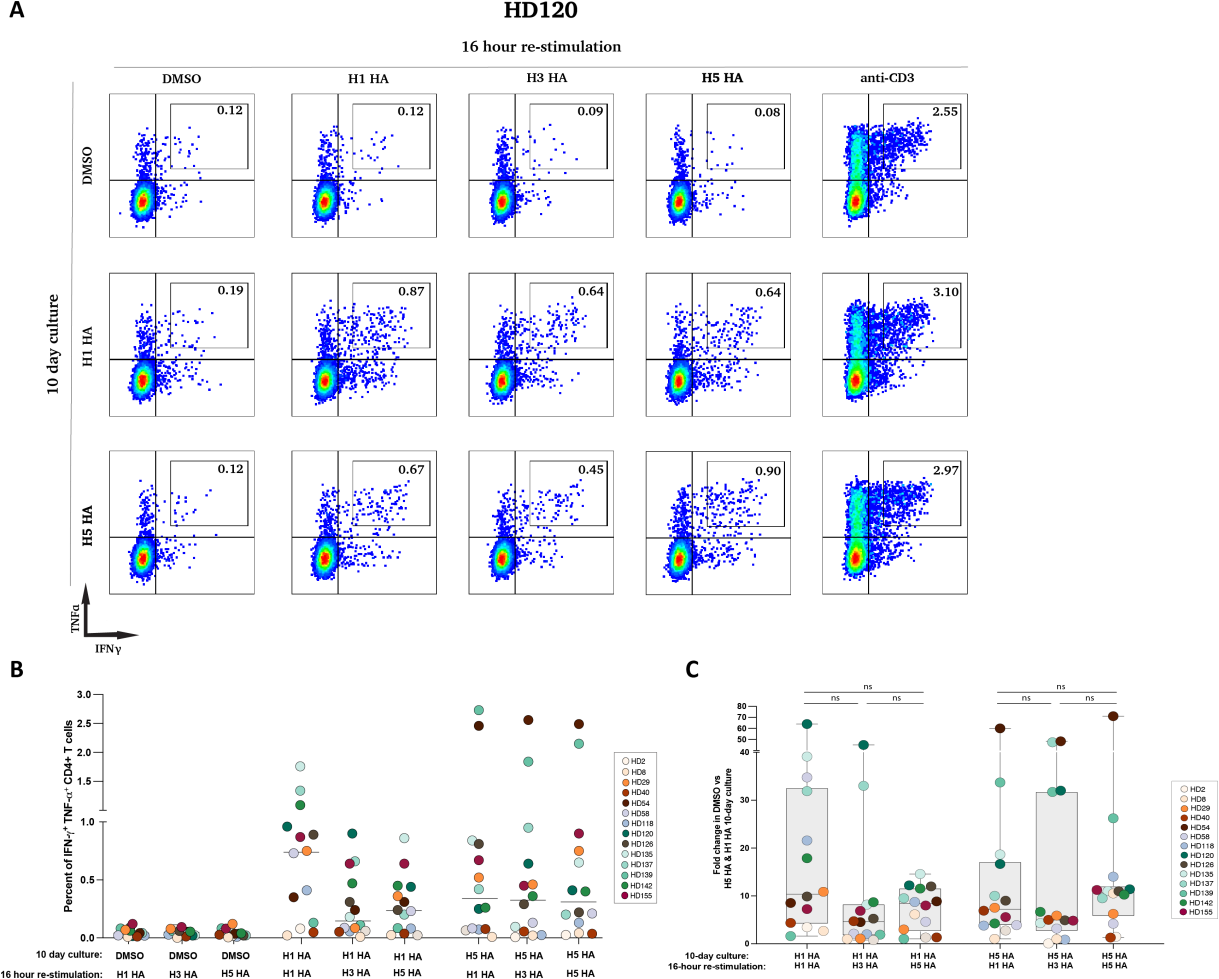
Cross-reactive responses between H1 HA and H5 HA-specific CD4+ T cells. **(A)** PBMCs from one participant (MP8) were pre-cultured for 10 days with either DMSO (top row) or H1 HA (middle row) or H5 HA peptides (bottom row). After expansion, cells were stimulated for 16 hours with DMSO, H1 HA peptides, H3 HA peptides, H5 HA peptides, or anti-CD3 antibodies and stained for IFN-γ and TNF-α expression. The percentages in each quadrant indicate the proportion of cells expressing the respective cytokines. **(B)** The percentage of IFN-γ+ TNF-α+ CD4+ T cells was measured in 14 participants after a 10-day pre-culture with H1N1 HA and H5N1 HA, and 16-hour restimulation with H1 HA, H3 HA, and H5 HA peptide pools, with DMSO as the negative control. **(C)** The fold change in IFN-γ+ TNF-α+ CD4+ T cells between DMSO and H1 HA or H5 HA after a 10-day culture in 14 participants is shown. Ns, not significant.

For the representative donor HD120, minimal responses were seen when cells were cultured with DMSO for 10 days and then stimulated with HA proteins. However, expanded H1 HA-specific CD4+ T cells showed robust cytokine responses after restimulation with H1, H3 and H5 HA, with IFN-γ+TNF-α+ cells detected at 0.87%, 0.64%, and 0.64%, respectively. Similarly, expanded H5 HA-specific T cells demonstrated strong responses, with percentages of IFN-γ+TNF-α+ cells at 0.67%, 0.45%, and 0.90% after restimulation with H1, H3, and H5 HA, respectively (**Figure 4A**).

Across all 14 donors, median percentages of IFN-γ+TNF-α+ T cells after H1 HA-expansion and restimulation with H1, H3, and H5 HA were 0.74%, 0.15%, and 0.24%, respectively. Similarly, median responses for H5 HA-specific T cells restimulated with H1 HA, H3 HA, and H5 HA were 0.34%, 0.325%, and 0.31%, respectively (**Figure 4B**).

The fold increase of antigen specific cells expanded in peptide pools was determined and revealed no significant differences in IFN-γ+TNF-α+ responses among the different HA restimulation conditions for either H1- or H5 HA-stimulated cells (**Figure 4C**).

### CD4^+^ TCR clonotypes cross-recognize H1 and H5 HA peptide pools

To further determine the extent of clonotypic cross-reactivity between H1 and H5 HA, the FEST assay was performed on CD8+ T cell-depleted PBMCs from three healthy donors following 10-day cultures with H1 and H5 HA peptide pools. The FEST assay captures antigen-driven expansion of CD4^+^ TCR clonotypes following peptide stimulation *in vitro* and can distinguish between mono-reactive and cross-reactive clonotypes. In a prior study, we cloned the alpha and beta chains of TCRs that were identified as being either mono-reactive or cross-reactive and demonstrated that Jurkat cells that were transduced with these receptors responded appropriately in functional assays (51). Antigen-specific TCRs were identified based on expansion relative to DMSO controls. Across all donors, both mono-reactive and cross-reactive clonotypes were detected.

**Figure 5:**
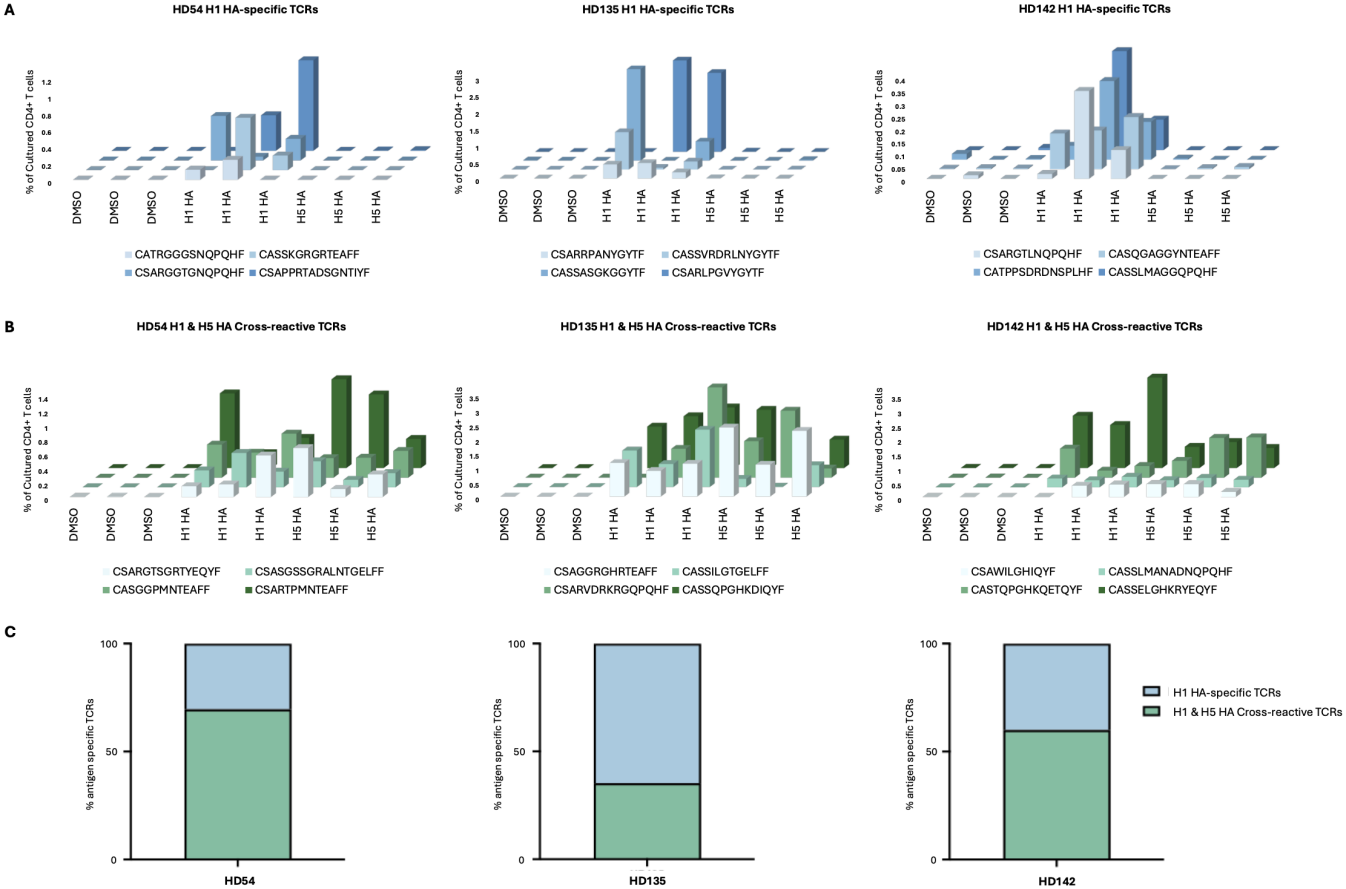
Identification of reactive CD4^+^ TCR clonotypes specific to H1N1 and H5N1 HA. After peptide culture, TCR Vβ CDR3 sequencing was performed to identify antigen-specific. CD4^+^ memory T cell clonotypes expanded using the FEST assay. Cross-reactive clonotypes were defined as those that expanded in response to both H1 HA and H5 HA peptide pools. H1 HA-specific clonotypes were defined as those that expanded in response to the H1 HA peptide pool only. Three technical replicates were performed for each peptide condition for three participants (HD54, HD135, HD142). **(A)** Frequency of representative H1 HA-specific TCR β clonotypes across DMSO, H1 HA, and H5 HA cultures for each participant. **(B)** Frequency of representative cross-reactive TCR β clonotypes across the same conditions. **(C)** Proportion of H1 HA-specific (blue) *vs*. cross-reactive (green) TCR clonotypes among all antigen-specific CD4^+^ T cells for each participant.

In donor HD54, 66.7% (16 of 23) of the clonotypes that recognized H1 HA cross-recognized H5 HA peptides. For HD135, 12 out of 34 clonotypes that recognized H1 HA also recognized H5 HA and for HD142, 6 out of 10 H1 HA-reactive clonotypes cross-recognized H5 HA peptides (**Figure 5**).

### Effector responses to H5N1 HA are limited and can be expanded by pre-culture with H1 or H5 HA proteins

**Figure 6:**
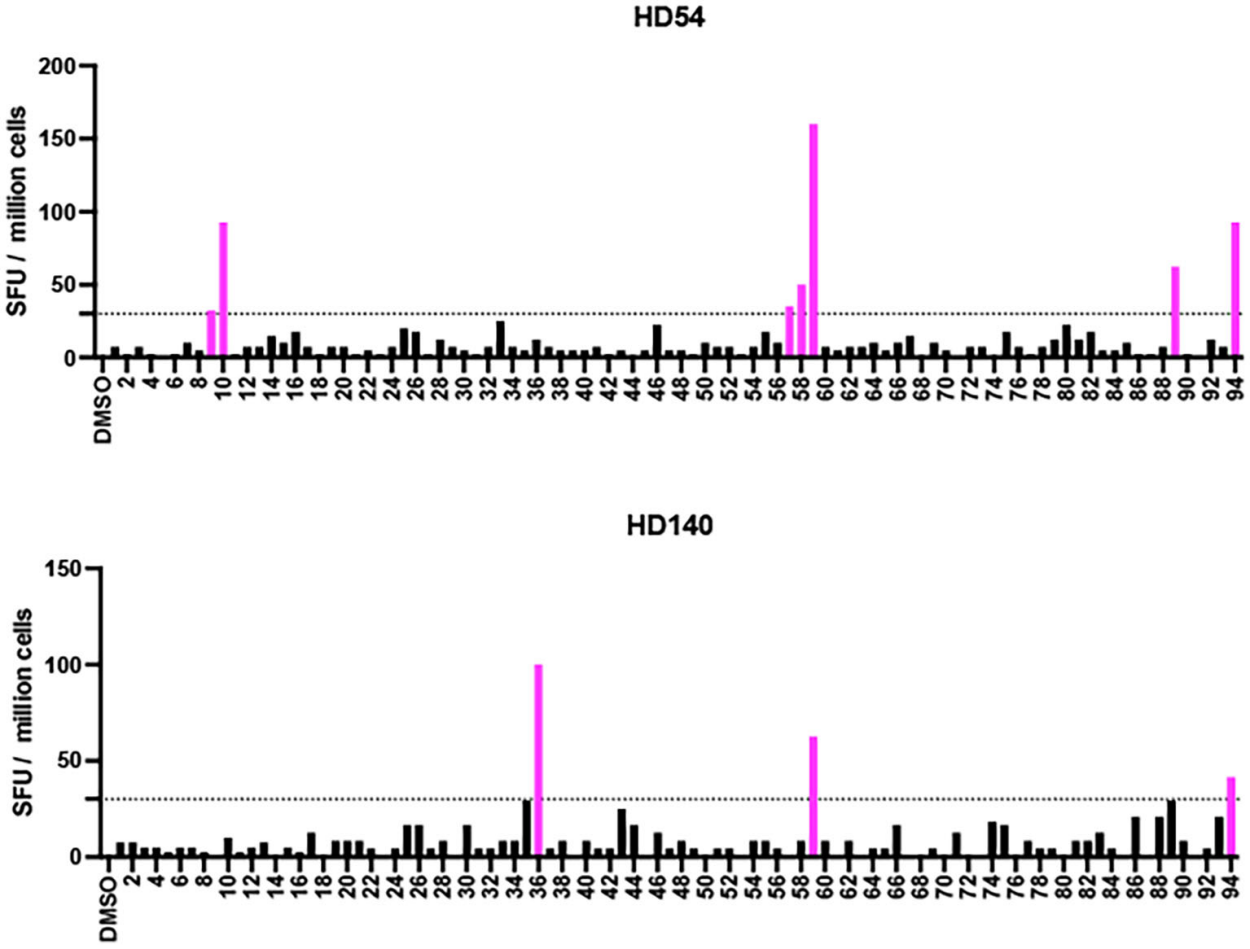
Identification of immunogenic H5N1 HA peptides across donors. The IFN-γ ELISpot assay was performed on samples obtained from 3 participants (HD36,HD54, and HD140). The SFU of PBMCs in response to each of the 94 overlapping H5N1 HA peptides are shown. Each data point represents the mean of 2 technical replicate values. The dotted horizontal line represents the threshold for positive responses (SFU ≥30). The pink vertical bars represent peptides that were targeted by each donor (SFU ≥30 and stimulation index ≥3). The percentages on the right represent the proportion of peptides targeted by each donor.

**Figure 7:**
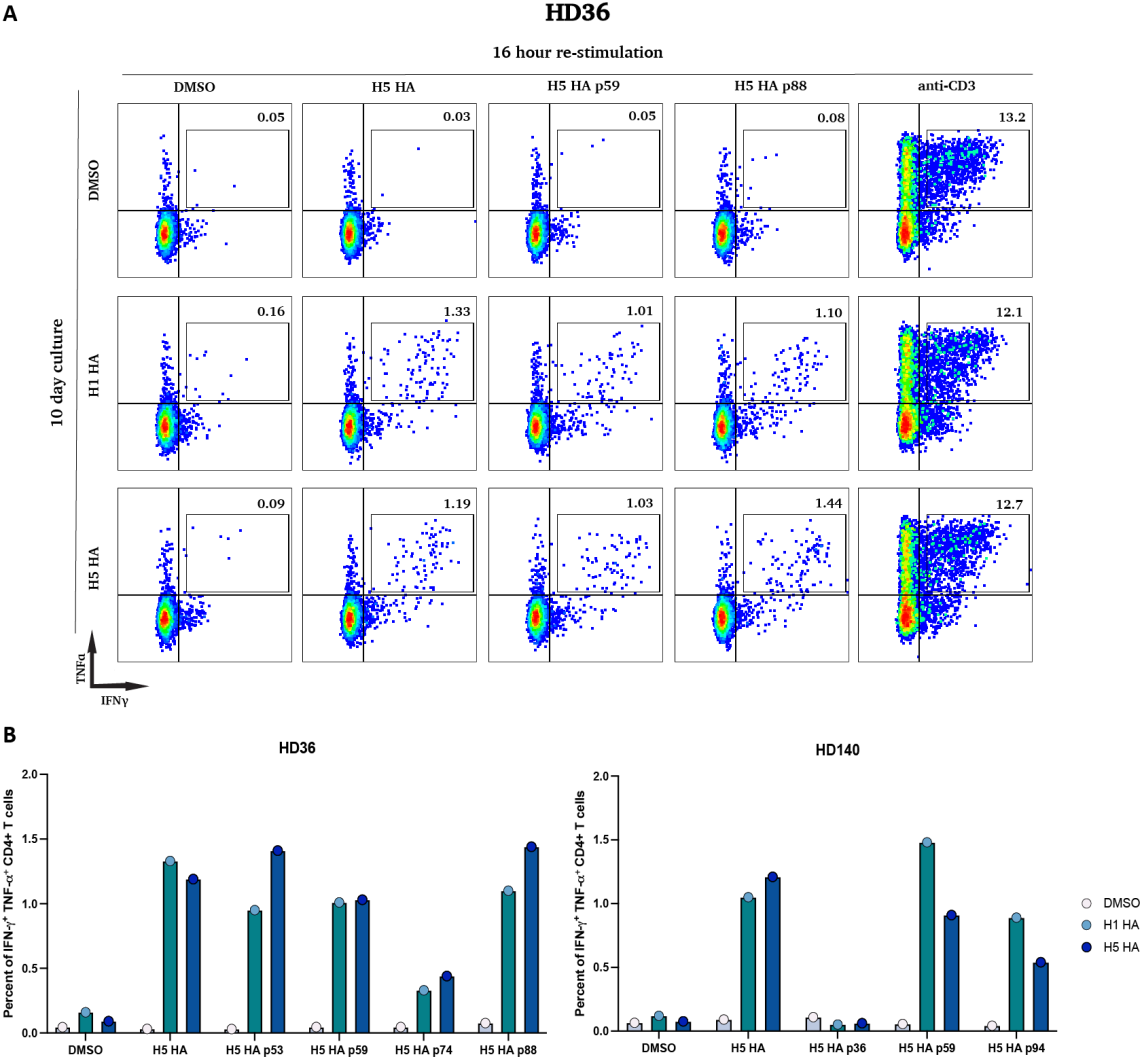
Shared H5N1 HA peptide targets of H1N1 HA- and H5N1 HA-expanded CD4^+^ T cells. **(A)** Representative flow cytometry plots from participant HD36. PBMCs were cultured for 10 days with DMSO (top row), H1N1 HA peptides (middle row), or H5N1 HA peptides (bottom row). On day 10, cells were restimulated for 16 hours with DMSO, full-length H5N1 HA peptide pool, individual H5N1 HA peptides (p59, p74, p88), or anti-CD3. Cells were stained for intracellular IFN-γ and TNF-α. Numbers in each quadrant represent the percentage of IFN-γ^+^ TNF-α^+^ CD4^+^ T cells. **(B)** Bar plots showing the percentage of IFN-γ^+^ TNF-α^+^ CD4^+^ T cells in two participants (HD36 and HD140) following 10-day cultures with DMSO, H1N1 HA, or H5N1 HA peptide pools, and 16-hour restimulation with DMSO, the full H5N1 HA pool, or individual peptides identified as immunogenic by ELISpot.

We identified 3 participants (HD36, HD54, HD140) who had detectable effector T cell responses to the H5 HA peptide pool in the ELISpot assay directly ex-vivo. A repeat ELISpot assay was performed using 94 overlapping peptides spanning the protein to determine the epitopes being targeted in these three participants. HD36 recognized 4 peptides, HD54 recognized 7 peptides and HD140 recognized 3 peptides (**Figure 6**). The degree of similarity between the targeted peptides and the analogous peptides in H1N1 HA ranged from 47.1-93.8% and in H3N2 HA ranged from 35.3-76.5% as shown in **Table 1**. To determine whether these responses were elicited by cross-reactive epitopes from H1 HA, PBMCs from HD36 and HD140 were cultured for 10 days with either H1N1 HA or H5N1 HA peptide pools and restimulated with the full H5 HA peptide pool or individual H5 HA peptides identified as targets by ELISpot. Cytokine production was measured by intracellular staining for IFN-γ and TNF-α. For HD36, H1 HA-expanded T cells responded to the full H5 HA peptide pool with 1.33% IFN-γ^+^TNF-α^+^ cells, closely matching the 1.19% observed in H5 HA-expanded cells. Responses to individual peptides were also comparable, with peptide 53 eliciting 0.95% *vs*. 1.41%, peptide 59 at 1.01% *vs*. 1.03%, peptide 74 at 0.33% *vs*. 0.44%, and peptide 88 at 1.01% *vs*. 1.44%, for H1- and H5-expanded cells respectively. For HD140 H1 HA-expanded T cells responded to the full H5 peptide pool with 1.05% IFN-γ^+^TNF-α^+^ cells, similar to the 1.21% observed in H5 HA-expanded cells. Likewise, both groups showed comparable responses to peptides 36 (0.05% *vs* 0.06%), 59 (1.48% *vs*. 0.91%) and 94 (0.89% *vs*. 0.54%) (**Figure 7**). The responses were all CD4 T cell-driven across both participants.

**Figure 8:**
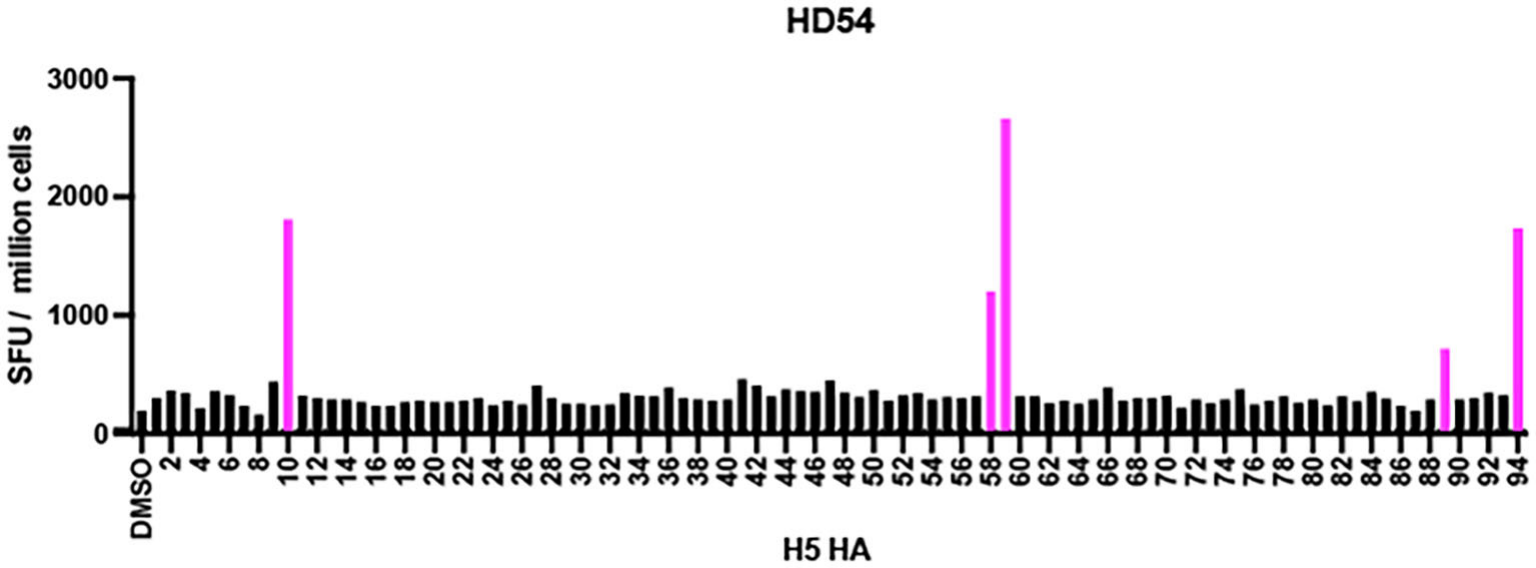
Identification of H5N1 HA peptides recognized by H1N1 HA-expanded CD4^+^ T cells. ELISpot assay results from two participants (HD54 and HD135) after a 10-day culture with H1N1 HA peptide pools, followed by restimulation with individual H5N1 HA peptides. Bars represent IFN-γ spot-forming units (SFU) per million cells for each of the 94 overlapping peptides spanning the H5N1 HA protein. DMSO was used as a negative control. Peptides that elicited a positive response (SFU ≥ 30 and stimulation index ≥ 3) are shown in pink. The percentages on the right represent the proportion of peptides targeted by each donor.

To determine whether culturing PBMCs from HD54 with H1 HA proteins would amplify any other H5 HA epitopes, a 10-day culture with H1 HA was performed and PBMCs were then restimulated with individual H5 HA epitopes. The only epitopes that were recognized were the 7 that were detected directly ex vivo (**Figure 8**).

## Discussion

This study highlights differences in T cell immunity to influenza and SARS-CoV-2 antigens. While nearly all participants responded robustly to S2S, only about half of the participants responded to H1 and H3 HA proteins. The low response to the HA proteins is notable as these laboratory workers received annual seasonal influenza vaccines and thus had repeated exposure to these antigens over many years. The lack of conserved epitopes in HA from different subtypes may partially explain why we did not see potent responses to the proteins we used in this study. In contrast, the majority of T cell epitopes in spike are not affected by mutations in SARS-CoV-2 variants (46). In addition to the higher frequency of circulating IFN-γ secreting T cells identified by the ELISpot assay, we also observed a higher frequency of S2S-specific memory cells in the expansion assay, and the FEST assay. There were also qualitative differences in cytokine profiles of expanded T cells with a higher percentage of H1 HA-specific T cells secreting IFN-γ-TNF-α-IL-2+ and a higher percentage of S2S-specific T cells secreting IFN-γ+TNF-α+IL-2-.

Emerging threats like highly pathogenic avian influenza H5 demand a better understanding of immune responses to guide vaccine strategies. Recent reports showing low mortality rates in H5 cases within the United States (6–9) prompted us to investigate T cell immunity to H5 in a cohort of influenza-vaccinated individuals. Cross-reactive T cell responses play a crucial role in providing immunity to emerging influenza strains, even in individuals without prior direct exposure. Roti et al. demonstrated that some healthy individuals with no known exposure to the virus harbor H5N1-specific CD4+ T cells. These responses were directed against MP1 and NP, and also HA. The H5 HA-specific responses were mostly cross-reactive with H1, H2, or H3 HA epitopes (29). Similarly, Babon et al. identified a conserved CD4+ T cell epitope in the HA fusion peptide that was recognized across multiple influenza A subtypes and even influenza B virus, suggesting that certain HA epitopes can elicit broad T cell immunity across diverse influenza strains (33). Our findings align with these studies, while we found that less than 20% of study participants had a detectable effector T cell response to H5 HA, we demonstrated that memory T cell responses could be found in the majority of individuals after *in vitro* expansion with either H1 or H5 HA. FEST analysis of clonotypic responses further supported this observation. Many antigen-specific CD4+ TCR clonotypes expanded in response to H1 or H5 HA were cross-reactive, with some individuals having more than half of their H1 reactive clonotypes responding to both peptide pools. These results indicate that H1-specific memory T cells frequently recognize conserved epitopes within H5 HA and can be expanded by either antigen. This expansion of cross-reactive T cells with H1 HA may partially explain why we saw a modest but significant increase in H5 HA-specific effector cells after immunization with seasonal influenza vaccine that contains H1 and H3 proteins. Interestingly, there was no difference in the magnitude of H5 HA memory responses after *in vitro* expansion with H1 versus H5 HA. This is likely because both HA proteins stimulated pre-existing cross-reactive T cells that were primed against H1 HA *in vivo* by vaccination and/or natural infection. These findings are consistent with a study showing that live attenuated H5N1 influenza virus preferentially expanded pre-existing seasonal H1 and H3-specific T cells rather than generating *de novo* H5 HA-specific responses (14).

In contrast to the inducible H5 HA memory responses that were present in many individuals, very few participants had detectable circulating effector T cell responses that targeted H5 HA. These T cell responses could be expanded with either H1 or H5 HA proteins suggesting that may due to cross-reactive responses to seasonal influenza viruses or vaccines rather than exposure to H5N1 viruses.

Mapping responses to individual H5N1 HA peptides confirmed the limited breadth of effector responses, with only a small fraction of the 94 peptides eliciting T cell activation. Despite this narrow targeting, H1N1 HA-expanded T cells were able to recognize shared H5N1 HA epitopes, and their cytokine profiles were functionally comparable to those of H5-expanded cells, indicating meaningful cross-reactivity. Notably, despite only 79.1% sequence similarity between the H1N1 and H5N1 HA proteins, we observed comparable cytokine responses to shared epitopes in both H1- and H5-expanded CD4^+^ T cells, underscoring the potential for functional cross-reactivity even across antigenically distinct strains.

Our study is limited by our focus on CD4+ T cell responses. Furthermore, cross-reactivity of only the HA protein was studied, and HLA typing of the participants was not performed so we were not able to predict the optimal epitopes targeted. We also did not measure responses to the more recent clade 2.3.4.4b H5N1 viruses. However, studies have shown a high degree of conservation of HA sequences in these viruses (53).

In conclusion, our study highlights key differences in T cell immunity to influenza and SARS-CoV-2 and we show a lower magnitude and frequency of influenza-specific responses, particularly against HA proteins. It is unclear whether these responses are due to the inherent immunogenicity of the viruses versus the different platforms used for vaccination. We demonstrated strong cross-reactivity among H1, H3, and H5 HA-specific T cells and identified TCR clonotypes that cross-recognize H1 and H5 peptide pools. We also provide evidence that pre-existing cross-reactive T cells that recognize H5 HA can be effectively expanded with H1 or H5 HA. Further studies will be needed to determine whether these cross-reactive responses provide protection against severe disease and whether mono-reactive H5-specific T cells can be elicited with H5N1 vaccines.

## Data Availability

The datasets presented in this study can be found in online repositories. The names of the repository/repositories and accession number(s) can be found in the article/**Supplementary Material**.
