# Supplementary figures and images for: T cell immunity to seasonal Influenza A and H5N1 viruses in laboratory workers receiving annual seasonal Influenza vaccines
Source: Front Immunol. 2025 Dec 18;16:1718805. doi: 10.3389/fimmu.2025.1718805 (PMC12756375; doi:10.3389/fimmu.2025.1718805)

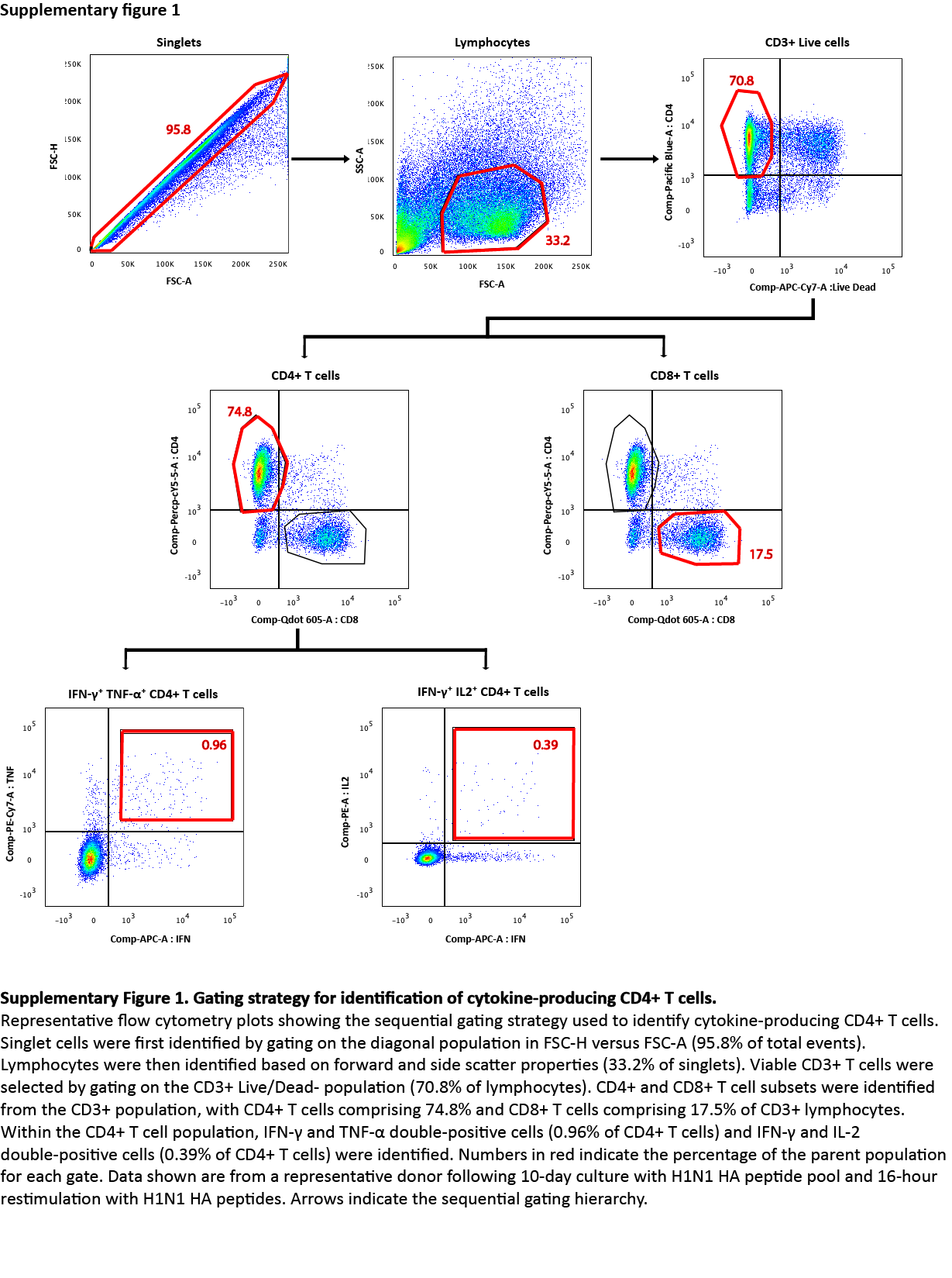

Supplement: Supplementary file 1 [file Image1.tif]

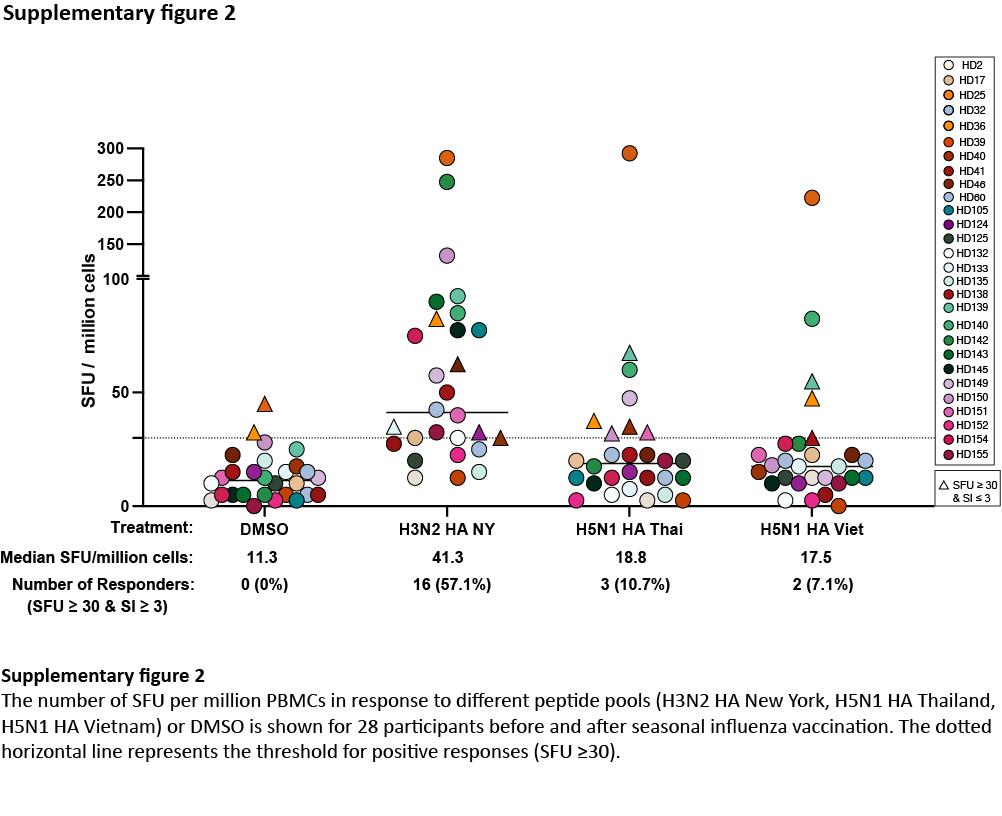

Supplement: Supplementary file 2 [file Image2.tif]

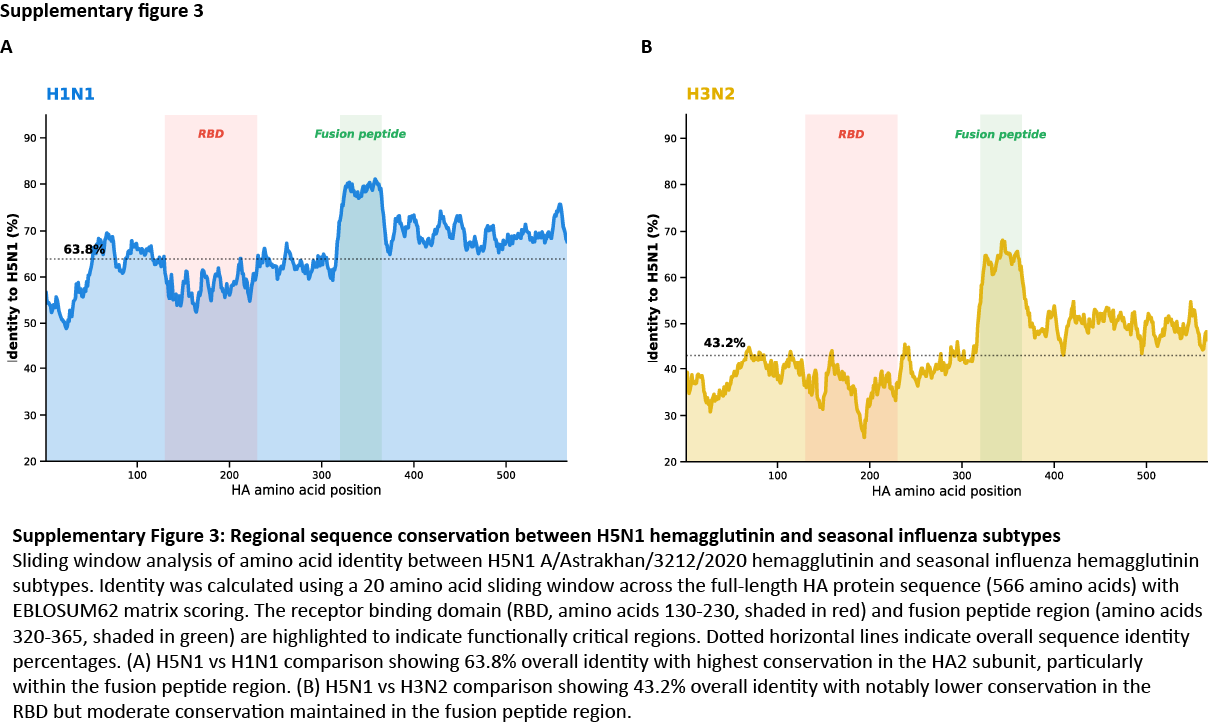

Supplement: Supplementary file 3 [file Image3.tif]
