# Supplementary material for: T cell immunity to seasonal Influenza A and H5N1 viruses in laboratory workers receiving annual seasonal Influenza vaccines
Source: Front Immunol. 2025 Dec 18;16:1718805. doi: 10.3389/fimmu.2025.1718805 (PMC12756375; doi:10.3389/fimmu.2025.1718805)
Supplement: Supplementary file 4 [file Table1.docx]

Supplementary Table 1. Demographics of study participants

| Participant | Sex | Age | Years since last COVID vaccine | Total number of COVID vaccines received | Years at Johns Hopkins Medicine* |
| --- | --- | --- | --- | --- | --- |
| HD2 | M | 41-50 | 3 | 4 | 10+ |
| HD8 | M | 51-60 | 2 | 4 | 10+ |
| HD14 | F | 31-40 | 2 | 4 | 8 |
| HD17 | M | 31-40 | 1 | 5 | 9 |
| HD18 | F | 41-50 | 2 | 5 | 8 |
| HD29 | F | 51-60 | 1 | 6 | 10+ |
| HD36 | M | 41-50 | 1 | 5 | 10+ |
| HD39 | M | 51-60 | 1 | 6 | 10+ |
| HD40 | M | 51-60 | 3 | 3 | 10+ |
| HD41 | M | 41-50 | 2 | 4 | 10+ |
| HD46 | F | 31-40 | 3 | 3 | 10+ |
| HD54 | F | 31-40 | 1 | 5 | 6 |
| HD58 | M | 21-30 | 1 | 5 | 7 |
| HD60 | M | 31-40 | 3 | 3 | 9 |
| HD75 | M | 21-30 | 1 | 3 | 6 |
| HD79 | F | 31-40 | 3 | 3 | 5 |
| HD81 | F | 21-30 | 2 | 4 | 1 |
| HD90 | F | 21-30 | 3 | 3 | 5 |
| HD120 | F | 21-30 | 1 | 4 | 1 |
| HD126 | F | 51-60 | 1 | 6 | 10+ |
| HD132 | M | 31-40 | 2 | 3 | 8 |
| HD133 | F | 21-30 | 2 | 3 | 2 |
| HD135 | M | 21-30 | NA | NA | 1 |
| HD137 | F | 21-30 | 1 | 5 | 1 |
| HD139 | F | 21-30 | 3 | 3 | 1 |
| HD140 | F | 21-30 | 3 | 2 | 4 |
| HD142 | M | 31-40 | 1 | 5 | 2 |
| HD143 | F | 21-30 | 1 | 3 | 1 |
| HD144 | M | 31-40 | 2 | 3 | 2 |
| HD145 | F | 21-30 | 2 | 4 | 4 |
| HD146 | M | 21-30 | 2 | 4 | 3 |
| HD147 | F | 31-40 | 3 | 2 | 1 |
| HD148 | F | 21-30 | 3 | 2 | 1 |
| HD149 | F | 21-30 | 3 | 2 | 2 |
| HD150 | F | 21-30 | 3 | 3 | 2 |
| HD151 | M | 21-30 | 1 | 3 | 2 |
| HD152 | M | 21-30 | 1 | 4 | 2 |
| HD154 | F | 21-30 | 2 | 4 | 1 |
| HD155 | F | 21-30 | 1 | 3 | 3 |
| HD156 | F | 21-30 | 1 | 4 | 1 |

*10+ years denotes 10 or more years

NA: Information not available
